# Robust data-driven segmentation of pulsatile cerebral vessels using functional magnetic resonance imaging

**DOI:** 10.1098/rsfs.2024.0024

**Published:** 2024-12-06

**Authors:** Adam M. Wright, Tianyin Xu, Jacob Ingram, John Koo, Yi Zhao, Yunjie Tong, Qiuting Wen

**Affiliations:** ^1^ Department of Radiology and Imaging Sciences, Indiana University School of Medicine, Indianapolis, IN, USA; ^2^ Weldon School of Biomedical Engineering Department, Purdue University, West Lafayette, IN, USA; ^3^ Department of Biostatistics and Health Data Science, Indiana University School of Medicine, Indianapolis, IN, USA

**Keywords:** cerebral vessel segmentation, cardiac pulsation, cerebral arteries, superior sagittal sinus, functional MRI (fMRI)

## Abstract

Functional magnetic resonance imaging (fMRI) captures rich physiological and neuronal information, offering insight into neurofluid dynamics, vascular health and waste clearance. Accurate cerebral vessel segmentation could greatly facilitate fluid dynamics research in fMRI. However, existing vessel identification methods, such as magnetic resonance angiography or deep-learning-based segmentation on structural MRI, cannot reliably locate cerebral vessels in fMRI space due to misregistration from inherent fMRI distortions. To address this challenge, we developed a data-driven, automatic segmentation of cerebral vessels directly within fMRI space. This approach identified large cerebral arteries and the superior sagittal sinus (SSS) by leveraging these vessels’ distinct pulsatile signal patterns during the cardiac cycle. The method was validated in a local dataset by comparing it to ground truth cerebral artery and SSS segmentations. Using the Human Connectome Project (HCP) ageing dataset, the method’s reproducibility was tested on 422 participants aged 36–90, each with four repeated fMRI scans. The method demonstrated high reproducibility, with an intraclass correlation coefficient > 0.7 in both cerebral artery and SSS segmentation volumes. This study demonstrates that large cerebral arteries and SSS can be reproducibly and automatically segmented in fMRI datasets, facilitating reliable fluid dynamics investigation in these regions.

## 1. Introduction

Functional magnetic resonance imaging (fMRI) can study various physiological and neuronal processes in the human brain [1–3]. Prior research has demonstrated the different physiological parameters influencing fMRI signals include vasomotion [4,5], respiration [6], cardiac pulsation [7,8] and blood flow and blood oxygenation [9]. These studies are typically performed in grey and white matter, often ignoring the upstream changes in the supplying arteries and downstream alterations in draining veins. With the growing interest in neurofluid dynamics and its associated waste clearance function, the dynamics of larger cerebral arteries and veins have become particularly relevant. Changes in fMRI signals within cerebral vessels reflect underlying physiological processes and may provide valuable insights into cerebral vascular health [10–12] as well as its coupling with cerebrospinal fluid (CSF) dynamics [13]. The availability of cerebral vessel segmentation could broaden fMRI research beyond grey matter to include upstream arteries and downstream veins.

Existing methods for cerebral vessel identification cannot accurately delineate these structures in the fMRI space. Time of flight (TOF) angiography and magnetic resonance venography (MRV) are used to identify cerebral arterial and venous structures, respectively [14]. However, large MRI databases often do not include these imaging procedures (e.g. Human Connectome Project [HCP], Alzheimer’s Disease Neuroimaging Initiative, UK Biobank) [15–18]. Without angiography scans, previous studies have leveraged the ratios of T1-weighted and T2-weighted anatomical scans to manually threshold the segmentations of the internal carotid artery and superior sagittal sinus (SSS) [10,12]. However, due to potential misregistration, these segmentations cannot accurately locate vessels in the fMRI images. fMRI scans are susceptible to echo-planar imaging-induced distortions [19], which affect both the Circle of Willis and venous sinus regions, leading to registration errors [20]. Given cerebral vessels' small size, even minor registration errors can misplace them into adjacent CSF or grey matter regions, which exhibit dramatically different dynamic patterns from vessels.

This challenge can be addressed by segmenting vessels directly in fMRI, eliminating the need for additional angiography scans and avoiding misregistration errors. One effective approach is to isolate vessel voxels by leveraging the unique signal dynamics present in these regions. Large cerebral arterial and venous blood demonstrate consistent pulsatile signals in cardiac-aligned fMRI, as demonstrated in previous studies. In 1999, Dagli *et al*. showed the presence of signal fluctuations near large cerebral vessels in retrospectively aligned fMRI to the cardiac cycle [21]. Henning Voss later revealed that hypersampled fMRI of large cerebral vessels reveals waveforms similar to peripherally measured pulse waves [22], and Aslan *et al*. extend this work by extracting cardiac fMRI waveforms in the absence of finger plethysmography recordings [23]. In 2023, Hermes *et al*. further demonstrated the high reproducibility of cardiac-induced pulsatility in fMRI signals near large cerebral vessels, including major cerebral arteries and the SSS [24]. Motivated by these findings, we developed a technique that automatically segments large cerebral arteries and the SSS by leveraging this pulsatile phenomenon.

This work aims to utilize the cardiac-induced pulsatility of fMRI signals to generate a data-driven automatic segmentation of large cerebral vessels. The automatic segmentation was tested on a local dataset and the HCP ageing cohort. In the local dataset, the automatic vessel segmentations were compared to ground truth references of the cerebral artery and SSS. Additionally, the HCP-aging dataset was utilized to demonstrate the method’s robustness and reproducibility on an extensive database spanning a participant age range of 36–90 years. This work reveals that the large cerebral arteries and the SSS can be reproducibly and automatically segmented in fMRI datasets.

## 2. Methods

### 2.1. Human participants

Two cohorts were used to complete this study: a local cohort (*n* = 5, age range: 21–36 years) and an ageing cohort from the HCP-ageing 2.0 Release (*n* = 714, age range 36–90 years). Both cohorts contained repeated fMRI scans. All local cohort participants provided written informed consent according to procedures approved by the Institutional Committee for the Protection of Human Participants at Indiana University or Purdue University. All HCP-ageing participants provided informed consent as outlined in Bookheimer *et al*. [15].

### 2.2. Imaging acquisition

Participants from both cohorts underwent imaging using a 3T Prisma Siemens scanner with a gradient strength of 80 mT/m and a slew rate of up to 200 T/m/s. The local cohort used a 64-channel head-neck coil, and the HCP-aging cohort used a 32-channel head-neck coil. Three MR sequences were used to complete this study, including a T1-weighted (T1w) anatomical scan, a resting-state fMRI scan, and a TOF scan. The TOF imaging was only acquired for the local cohort. Simultaneous finger plethysmography (PPG) was recorded during all fMRI scans and used as a reference for retrospective cardiac alignment.

The fMRI acquisition parameters for local participants were repetition time (TR) = 366 ms, echo time (TE) = 29.80 ms, flip angle (FA) = 35°, voxel size = 2.5 × 2.5 × 2.5 mm³, volumes = 500, multiband factor = 8 and an acquisition time = 3.05 min. All repeated local fMRI scans were acquired with anterior-posterior (AP) phase encoding. The fMRI acquisition parameters for HCP-aging participants were TR = 800 ms, TE = 37.0 ms, FA = 52°, voxel size = 2.0 × 2.0 × 2.0 mm³, volumes = 488, multiband factor = 8 and an acquisition time = 6.51 min. The repeated fMRI scans in the HCP-ageing cohort were acquired over two imaging sessions (separate days). For each session, a pair of scans with opposite phase encoding directions were acquired (AP and posterior-anterior (PA)).

The T1w anatomical imaging data were collected using a three-dimensional magnetization rapid gradient echo (MPRAGE) sequence. The acquisition parameters in local participants were TR = 2300.0 msec between inversion pulses, 7.1 ms between excitation pulses (208 excitations per inversion pulse), inversion time (TI) = 900 ms, TE = 2.98 ms, FA = 9°, voxel size = 1.0 × 1.0 × 1.0 mm³ and an acquisition time = 5.5 min. The acquisition parameters for HCP-ageing cohorts were TR = 2500.0 ms between inversion pulses, TI = 1000 ms, TE = 2.22 ms, FA = 8°, voxel size = 0.8 × 0.8 × 0.8 mm³ and an acquisition time = 8.37 min. The acquisition parameters for the TOF scans in local participants were TR = 21.0 ms, TE = 3.42 ms, FA = 18°, voxel size = 0.3 × 0.3 × 0.6 mm³, slice thickness = 40 mm with a 2 mm gap and an acquisition time = 4.72 min.

### 2.3. Participant image quality criteria

Each fMRI dataset was checked for finger plethysmography signal quality and motion artefacts. We developed an in-house function to automatically assess the quality of the finger plethysmography based on the percentage of signal power centred around the cardiac frequency (see electronic supplementary material, figures S1-S3). fMRI data were excluded if the finger plethysmography signal was inadequate or contained motion artefacts (FSL: MCFLIRT [25], maximum translation > voxel dimension).

### 2.4. Image processing

#### 2.4.1. fMRI pre-processing

The initial ten volumes of the fMRI were excluded to reduce T1-relaxation effects. A voxel-wise high-pass Butterworth filter with a cutoff frequency of 0.005 Hz was then applied to remove the fMRI signal’s DC offset. Although flipped phase-encoding data were available, distortion correction was not performed because it could disrupt the slice timing profile due to data interpolations between adjacent slices with different readout times.

#### 2.4.2. General vessel region identification

**Figure 1.**
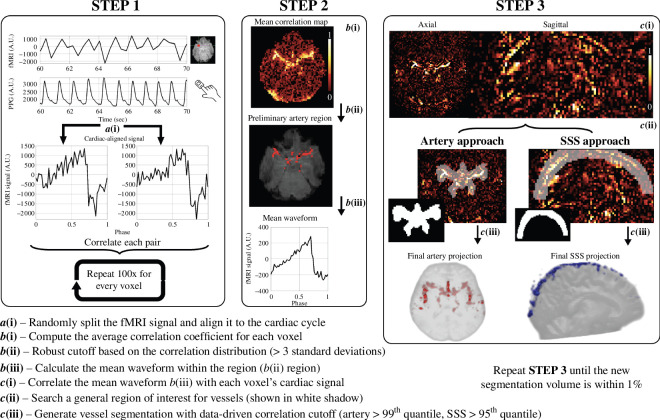
Schematic overview of the data-driven segmentation pipeline. (*a*(i)) Time-detrended single voxel fMRI time series (top), the corresponding finger plethysmography signal (middle) and cardiac-aligned signal pair from an arterial voxel (bottom). (*b*(i)) Mean correlation map computed from the 100 signal pair correlations completed in Step 1. (*b*(ii)) Preliminary artery region generated by identifying voxels with correlations three standard deviations above the mean. (*b*(iii)) The mean waveform is based on the voxels in the preliminary artery region. (*c*(i)) Correlation map between the mean waveform of the preliminary artery region and the voxel-wise cardiac-aligned fMRI signal. (*c*(ii) left) General artery ROI overlay with the correlation map (*c*(ii) right) and general SSS ROI overlay with the correlation map. (*c*(iii) left) The final artery segmentation (displayed as a three-dimensional projection) is defined as voxels within the general artery ROI. (*c*(iii) right) The final SSS segmentation (displayed as a three-dimensional projection) is defined as voxels within the general SSS ROI. Step 3 employs an iterative approach to stabilize the segmentation volume within 1% that is detailed in the methods.

An initial overly inclusive cerebral artery and SSS region were generated to define a general search region in the data-driven vessel segmentation. These search regions are referred to as the general cerebral artery region of interest (ROI) and general SSS ROI. The general cerebral artery ROI was derived from the statistical brain atlas Dunås *et al*. developed using manually labelled four-dimensional flow MRI [26]. The atlas was modified to only include the anterior cerebral artery (ACA), middle cerebral artery (MCA) and posterior cerebral artery (PCA) and their major branches. Using the atlases probability maps of these arteries, a three-dimensional maximum intensity projection was generated. The three-dimensional maximum intensity projection in MNI space was then dilated (Matlab: imdilate, kernel size = 3) and binarized by selecting all voxels with a probability greater than 0.5%, resulting in an overly inclusive ROI around the major cerebral arteries. The PCA region was additionally modified by making a coronal cut at the midbrain level to reduce the potential of including the quadrigeminal cistern (CSF space) and the great cerebral vein of Galen. A general SSS ROI was generated by manually segmenting the SSS on the MNI152 T1 1 × 1 × 1 mm^3^ brain, which was later dilated in the subject’s fMRI space to make it overly inclusive. Subsequently, the statistical brain atlas and manual SSS segmentation were transformed from the MNI space to the T1w space (ANTS: antsApplyTransforms, nonlinear interpolation) and then to the fMRI space (FSL: FLIRT, nearest-neighbour interpolation). Following the SSS mask transformation into fMRI space, it was dilated (Matlab: imdilate, kernel size = 3) to ensure the inclusiveness of the general SSS ROI. Representative examples of the general artery and SSS ROIs in fMRI space are displayed in the lower left panels of figure 1*b*(ii) and electronic supplementary material, figure S4.

### 2.5. Data-driven vessel segmentation generation

The data-driven vessel segmentation is a multi-step process that includes three significant steps. These steps are summarized in figure 1 and include a randomized realignment of the fMRI data to the cardiac cycle (Step 1), the generation of a preliminary artery region (Step 2) and the generation of a finalized segmentation for both cerebral arteries and the SSS (Step 3).

#### 2.5.1. Randomized fMRI signal cardiac alignment (Step 1)

The preprocessed fMRI signals were randomly split into two temporal subsets and retrospectively aligned to the cardiac cycle, resulting in a voxel-wise cardiac-aligned signal pair (figure 1*a*(i)). The splitting was completed by randomly selecting half of the time points of the time series using (Matlab: randperm) to represent the first temporal set. The second temporal set comprised all time points not used in the first. After selecting the time points, each temporal set was realigned to the cardiac cycle using finger plethysmography as the reference. Then, a voxel-wise correlation coefficient between each cardiac-aligned signal pair was calculated using equation (2.1):


(2.1) $$\rho \left(A,B\right)=\frac{1}{N-1}\sum_{\varphi =1}^{N}\left(\frac{A_{\varphi }-\mu_{A}}{\sigma_{A}}\right)\;\left(\frac{B_{\varphi }-\mu_{B}}{\sigma_{B}}\right)$$

where *N* was the length of the cardiac-aligned signals *A* and *B*, φ was the cardiac phase, *μ* and *σ* were the mean and standard deviation of the respective signal. The process of generating cardiac-aligned signal pairs and their correlation coefficient was repeated one hundred times.

#### 2.5.2. Preliminary artery region (Step 2)

A voxel-wise mean correlation map was generated by taking the mean of the 100 repeated correlation coefficients (figure 1*b*(i)). A higher correlation coefficient indicated an increased likelihood that the voxel contains a vessel due to the strong pulsatility [24]. Considering the correlation coefficients varied among participants, a data-driven approach was utilized to identify a participant-specific correlation threshold to identify voxels that are highly likely to contain large vasculature. The threshold was generated as the correlation coefficient three standard deviations above the mean of all brain voxels (T_preliminary_). Once this threshold was generated, all voxels within the general cerebral artery ROI with mean correlation coefficients greater than T_preliminary_ were considered probable vascular voxels. This process produces the preliminary artery region that is later refined (figure 1*b*(ii)). The preliminary artery region represented highly pulsatile cardiac-aligned fMRI signals, mainly consisting of actual vessels with some spurious voxels. The mean waveform of this preliminary artery region was then calculated to represent the signal waveform of the arterial region (figure 1*c*(iii)). This waveform was used to refine future cerebral artery and SSS segmentations.

#### 2.5.3. Refined vessel segmentation (Step 3)

The final step utilized an iterative approach to finalize the artery and SSS segmentations (slight differences in SSS segmentation finalization will be noted in parentheses). First, a new correlation map was generated by calculating a voxel-wise correlation between the cardiac-aligned fMRI signal and the mean waveform from the preliminary artery region (figure 1*c*(i)). Second, a data-driven correlation cutoff was generated based on the 99^th^ quantile of correlations within the brain but outside the general artery and general SSS ROIs (95^th^ quantile for the SSS). Third, all voxels within the general artery ROI (figure 1*c*(ii) left) exceeding the data-driven threshold were identified as the current vessel segmentation (the general SSS ROI was used for SSS segmentation, and all brain voxels were excluded, figure 1*c*(ii) right). These three steps were repeated until the segmentation volume was stabilized to within 1% of the previous iteration’s segmentation volume. When an iteration was necessary, a new correlation map was generated using the mean cardiac-aligned fMRI waveform from the most recent vessel segmentation. The vessel segmentations were complete once the segmentation volume was stabilized (figure 1*c*(iii)).

### 2.6. Performance assessment and reproducibility

#### 2.6.1. Qualitative comparison with ground truth

Ground truth artery regions were generated using TOF scans. Manually set intensity thresholds were used to binarize TOF scans, which were then quality-checked to ensure proper segmentations of the PCA, ACA and MCA and their major branches. The binarized TOF scans were transformed into T1w space (FSL: FLIRT, nearest-neighbour interpolation) and then to fMRI space (FSL: FLIRT, nearest-neighbour interpolation). Once in fMRI space, the TOF binary mask was limited to voxels within the general cerebral artery ROI (to ensure the overall segmentation region matched the one used in the data-driven segmentation) and was defined as the arterial ground truth segmentation. Ground truth SSS were manually drawn on T1w images and were transformed into fMRI space (FSL: FLIRT, nearest-neighbour interpolation). Once in fMRI space, the manually drawn SSS segmentation was limited to voxels within the general SSS ROI (to ensure the overall segmentation region matched the one used in the data-driven segmentation) and was defined as the SSS ground truth segmentation.

The data-driven artery segmentation results of the local participants were qualitatively compared with the corresponding ground truth artery segmentation for the five local participants (the HCP-aging dataset does not have TOF scans). The SSS segmentation results of both local and HCP participants were qualitatively compared with the corresponding ground truth SSS segmentation.

#### 2.6.2. Quantitative comparison with ground truth

For all local participant datasets, the Dice score and percent overlap were calculated to compare the data-driven segmentation for both artery and SSS to the corresponding ground-truth segmentations. The Dice score was computed using equation (2.2) (Matlab: dice):


(2.2) $$Dice=\;\frac{2\;\left|X\;\cap \;Y\right|}{\left|X\right|+\;\left|Y\right|}$$

where X represented the data-driven segmentation and Y represented the ground-truth segmentation. The percent overlap was calculated as the total number of voxels overlapping a reference segmentation using equation (2.3):


(2.3) $$Overlap\;\left(\%\right)=\;\frac{SV}{RV}*100$$

where SV represented the number of voxels in the tested segmentation, while RV represented the number of voxels in the reference segmentation. The overlap was computed for all scenarios, which allowed both the data-driven and ground-truth segmentation to serve as the reference segmentation.

#### 2.6.3. Reproducibility assessment

The reproducibility of the data-driven segmentation was assessed by calculating the intraclass coefficient (ICC, two-way mixed effects, single rater, absolute agreement) of the segmentation volumes. Segmentation volumes were compared between all possible unique scan combinations (four repeated scans resulted in six comparisons). Scatter plots with a total least square regression were used to visualize the correlation between segmentation volumes. Person correlation was used to test the strength of the linear correlation between segmentation volumes. Bland–Altman plots were used to visualize the agreement between segmentation volumes. A paired two-tailed *t*‐test assuming equal variance was used to test the agreement between segmentation volumes (*p* < 0.05 indicated the segmentation volumes were unequal). *p*<0.05 was considered statistically significant.

## 3. Results

### 3.1. Participant inclusion and exclusion

Participants with all four fMRI repeats that passed quality checks were included in the analysis. Examples of inadequate finger plethysmography data were summarized in electronic supplementary material, figures S1-3. No local participants were excluded from the analysis due to careful monitoring of finger plethysmography signals during data collection. We excluded 297 HCP participants from the analysis; 101 had at least one of the scans without a plethysmography recording, 13 were excluded for both poor plethysmography signal and excessive motion, 19 were excluded for excessive motion and 164 were excluded for poor plethysmography signal. Following data quality control, 5 local participants and 417 HCP-ageing participants were included in the analysis.

### 3.2. Data-driven and ground truth segmentation results aligned

**Figure 2.**
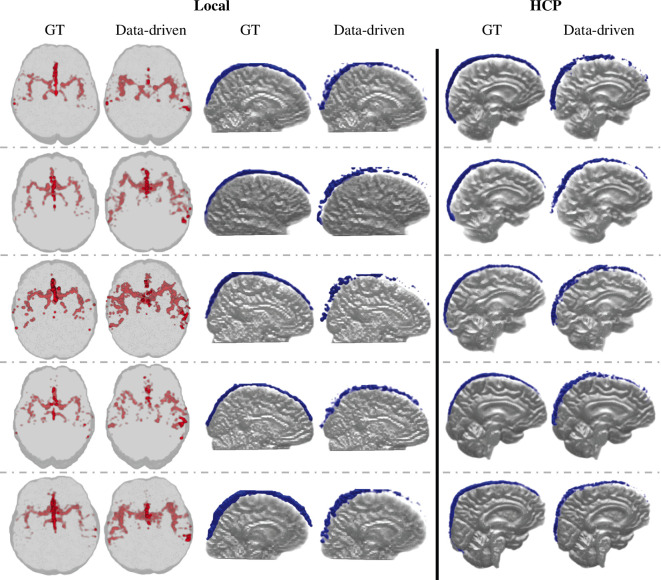
Three-dimensional projections of fMRI-derived vessel segmentations (data-driven, right of pair) compared with ground truth (GT, left of pair) in fMRI space (Artery—Axial view; SSS—Sagittal view). The local participant’s data-driven artery segmentations are compared with TOF intensity-based artery segmentations (left columns), and the data-driven SSS segmentations are compared with manual SSS segmentations (middle columns). The five representative HCP participants’ data-driven SSS segmentations are compared with manual SSS segmentations (right columns).

**Figure 3.**
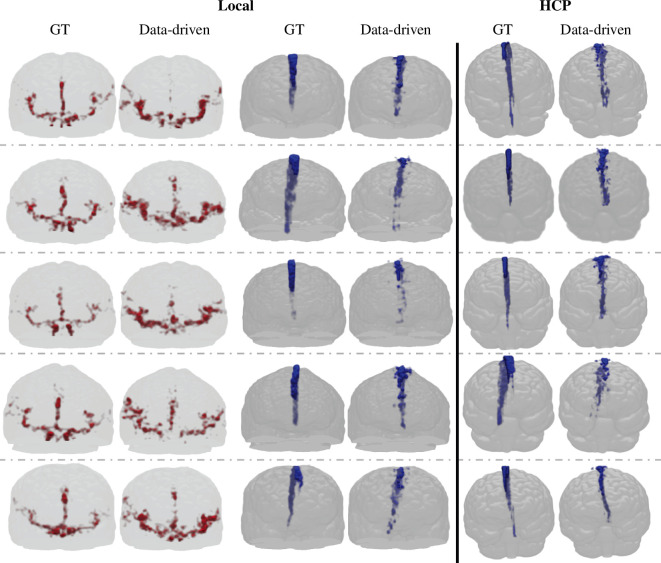
Coronal three-dimensional projections of fMRI-derived vessel segmentations (data-driven, right of pair) compared with ground truth (GT, left of pair) in fMRI space. The local participant’s data-driven artery segmentations are compared with TOF intensity-based artery segmentations (left columns), and the data-driven SSS segmentations are compared with manual SSS segmentations (middle columns). The five representative HCP participants’ data-driven SSS segmentations are compared with manual SSS segmentations (right columns).

The ground truth (left of image pair) and data-driven (right of image pair) segmentations of five local and five HCP-aging participants were summarized in figures 2 and 3. All segmentations were displayed as projections and overlaid onto T1-weighted images in fMRI space (figure 2, arteries—axial view, SSS—sagittal view; figure 3, arteries and SSS—coronal view). Qualitatively, the data-driven artery segmentation matched closely with the ground truth segmentations derived from TOF images (figures 2 and 3—left columns). The Circle of Willis and its components were distinguishable and comparable to the TOF references in all participants. In the local (figures 2 and 3—middle columns) and HCP (figures 2 and 3—right columns) participants, the SSS segmentation results showed a continuous vessel structure from the anterior to the posterior in the three-dimensional projection and closely aligned with the manually segmented ground truth.

In fMRI space, the Dice score (mean ± s.d.) between the data-driven artery and TOF segmentations were 0.11 ± 0.03, and the data-driven SSS and SSS ground truth segmentations were 0.28 ± 0.08 (electronic supplementary material, figure S5). The mean artery segmentation volumes were 9.53 ± 2.09 cm^3^ and 5.61 ± 1.33 cm^3^ in the data-driven and TOF segmentation. The mean SSS segmentation volumes were 4.70 ± 1.59 cm^3^ and 5.83 ± 0.95 cm^3^ in the data-driven and ground-truth segmentation (electronic supplementary material, figure S6). The low Dice score is attributed to misregistration between TOF and fMRI images caused by EPI distortions in fMRI. The ground-truth segmentations were commonly displaced from the data-driven segmentation by a single voxel or more (electronic supplementary material, figure S7).

### 3.3. Data-driven segmentations were reproducible

**Figure 4.**
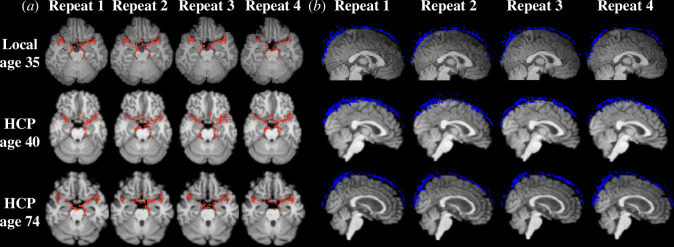
Representative data-driven artery and SSS segmentations of repeated scans. The three representative participants included were a local participant (35 years, top), one younger HCP participant (40 years, middle), and one older HCP participant (74 years, bottom). (*a*) A single axial slice artery segmentation (red) includes portions of the middle cerebral and posterior cerebral arteries. (*b*) A single sagittal slice SSS segmentation (blue).

The data-driven segmentation of repeated scans for a local participant (age 35), a younger HCP participant (age 40) and an older HCP participant (age 74) were summarized in figure 4. For each repeated scan, one axial slice of the artery segmentation (red) was overlaid onto T1-weighted MR images (figure 4*a*), and one sagittal slice of the SSS segmentation (blue) was overlaid onto T1-weighted MR images (figure 4*b*). The qualitative comparison of the four repeated scans demonstrates that the data-driven artery and SSS segmentations performed consistently across all repeats. The single-slice views display some discontinuity in the segmentation that was not visible when viewing the segmentations as a projection.

**Figure 5.**
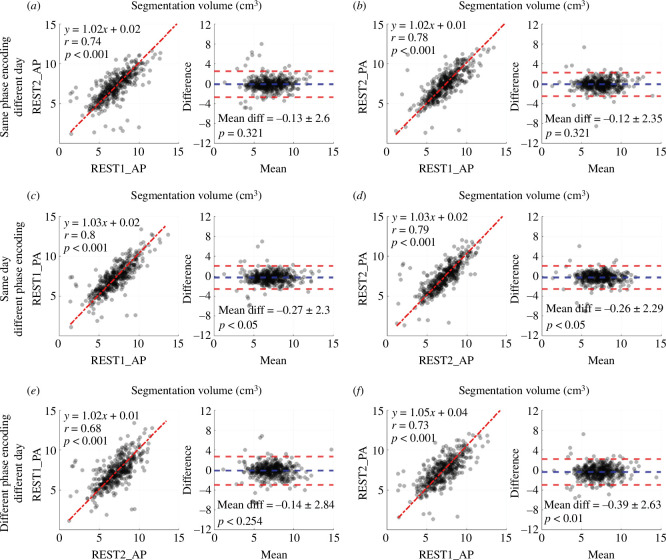
Assessment of data-driven artery segmentation volume (units: cm^3^) reproducibility between scans with different phase-encoding directions (AP, anterior-posterior; PA, posterior-anterior) and scan days (REST1 and REST2) in all local and HCP participants (*n* = 422). The comparisons include the same phase encoding direction and different scan day (*a,b*), same scan day and different phase encoding direction (*c,d*) and different phase encoding direction and different scan day (*e,f*). Within each lettered panel, Left: Linear regression with total least-squares fit and Pearson correlation. Right: Bland-Altman plot (difference calculated as the x-axis minus y-axis of the respective regression on the left) and a paired *t*‐test between segmentation volumes (*p* < 0.05 indicated the segmentation volumes were unequal).

**Figure 6.**
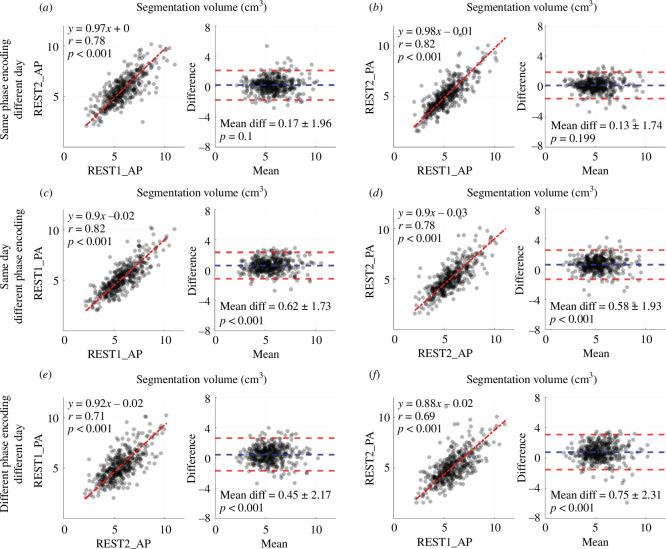
Assessment of data-driven SSS segmentation volume (units: cm^3^) reproducibility between scans with different phase-encoding directions (AP, anterior-posterior; PA, posterior-anterior) and scan days (REST1 and REST2) in all local and HCP participants (*n* = 422). The comparisons include the same phase encoding direction and different scan day (*a*, *b*), same scan day and different phase encoding direction (*c*, *d*) and different phase encoding direction and different scan day (*e*, *f*). Within each lettered panel, Left: Linear regression with total least-squares fit and Pearson correlation. Right: Bland–Altman plot (difference calculated as the x-axis minus y-axis of the respective regression on the left) and a paired *t*‐test between segmentation volumes (*p* < 0.05 indicated the segmentation volumes were unequal).

All local and HCP participants with four scans that passed the quality assessment step were used in a reproducibility assessment (*n* = 422). The repeated artery segmentation volumes had an ICC of 0.751 (95% CI: 0.717–0.783, *p* < 0.001). The repeated SSS segmentation volumes had an ICC of 0.725 (95% CI: 0.693–0.786, *p* < 0.001). The segmentation volume reproducibility for all possible scan combinations was illustrated for artery segmentations in figure 5 and SSS segmentations in figure 6.

The artery segmentation volumes from scans completed with the same phase encoding on different days showed strong correlation and good agreement (figure 5*a*,*b*: AP: *y* = 1.02*x* + 0.02, *r* = 0.74, *p* < 0.001; mean diff = −0.13 ± 2.6 cm^3^, *p* = 0.321; PA: *y* = 1.02*x* + 0.01, *r* = 0.78, *p* < 0.001; mean diff = −0.12 ± 2.35 cm^3^, *p* = 0.321). The volumes for scans completed on the same day with different phase-encoding directions showed strong correlations, but the mean volume differed between scans (figure 5*c*,*d*, see figure for statistics). The volumes for scans completed on different days and different phase-encoding directions showed strong correlations, but the mean volume differed between the REST2_PA vs. REST1_AP scans (figure 5*e*,*f*, see figure for statistics). The slope greater than one and negative mean difference for all scans with different phase-encoding directions indicated that the PA artery segmentation volumes were typically larger than AP volumes.

The SSS segmentation volumes from scans completed with the same phase encoding on different days showed strong correlation and good agreement (figure 6*a*,*b*: AP: *y* = 0.97*x* + 0.0, *r* = 0.78, *p* < 0.001; mean diff = 0.17 ± 1.96 cm^3^, *p* = 0.1; PA: *y* = 0.98 *x*-0.01, *r* = 0.82, *p* < 0.001; mean diff = 0.13 ± 1.74 cm^3^, *p* = 0.199). The volumes for scans completed on the same day with different phase-encoding directions showed strong correlations, but the mean volume differed between scans (figure 6*c*,*d*, see plot of statistics). The volumes for scans completed on different days and different phase-encoding directions showed strong correlations, but the mean volume differed between scans (figure 6*e*,*f*, see plot of statistics). The slope of less than one and positive mean difference for all scans with different phase-encoding directions indicated that the AP SSS segmentation volumes were typically larger than PA volumes.

### 3.4. Group averaged cerebral artery and SSS segmentations in MNI space

**Figure 7.**
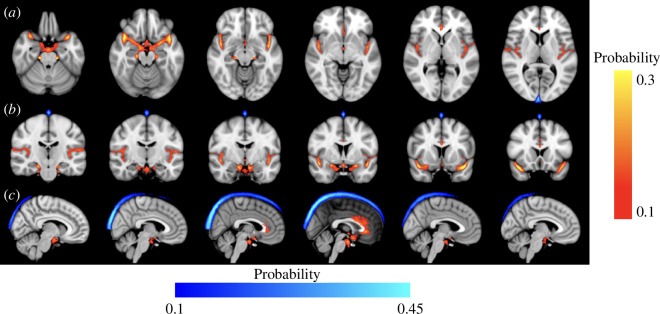
HCP participants’ average probability of large cerebral arteries and superior sagittal sinus segmentations scans with phase encoding in the anterior-posterior direction in MNI152 T1 1 mm isotropic space (*a*—Axial, *b*—Coronal, *c*—Sagittal).

Due to differences in segmentation volume between scans with different phase-encoding directions, only scans with the same phase-encoding direction were averaged in MNI space. The large cerebral artery and SSS segmentations for all HCP participants’ scans were transformed into the MNI152 T1 1 mm space and averaged to create a group probability map of the arteries and SSS for all AP phase encoding direction was summarized in figure 7. The PA phase encoding direction was summarized in electronic supplementary material, figure S8. The highest observed probability in the artery space was approximately 30% and observed near the ascending portion of MCA-m1 before the MCA bifurcation (figure 7*b*—right three panels). The MCA-m1 segment had higher probabilities than the segments of the ACA and PCA. The highest probability in the SSS space was approximately 50% and observed near the posterior portion of the SSS (figure 7*c*). The higher artery segmentation volume observed in the PA phase encoding scans presented as a stretched MCA in the anterior direction compared to the MCA in the AP phase encoded scan (electronic supplementary material, figure S9).

### 3.5. Spectral analysis confirmed that data-driven segmentation pinpoints voxels with high cardiac pulsatility

**Figure 8.**
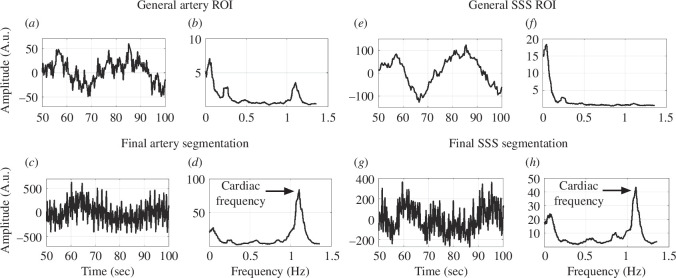
A representative participant’s progression of the mean fMRI time series and frequency response from the start of the segmentation process using the general vascular ROI to the end of the data-driven segmentation (final vascular segmentation). In this scan, the fMRI repetition time was 0.366 s (Nyquist frequency = 1.366 Hz), and the participant’s average heart rate was 67 beats per min (cardiac frequency = 1.117 Hz). The general artery ROI mean time series (*a*) and frequency spectrum (*b*). The final artery segmentation mean time series (*c*) and frequency spectrum (*d*). The general SSS ROI mean time series (*e*) and frequency spectrum (*f*). The final SSS segmentation mean time series (*g*) and frequency spectrum (*h*).

Data-driven segmentation was a multi-step process that began with a general vessel ROI and was finalized with vascular segmentation. In a representative subject, the mean fMRI real-time signal and the corresponding frequency response for both the general vascular ROI and the final vascular segmentation were summarized in figure 8. The general artery ROI time series contained some high-frequency fluctuations (figure 8*a*), however, the magnitude of this fluctuation was heavily amplified in the final artery segmentation (figure 8*c*). This observation was further supported by the increase in the amplitude of the cardiac frequency (~1 Hz) from the general artery ROI (figure 8*b*) to the final artery segmentation (figure 8*d*). Similar findings were observed in the SSS segmentation results. The time series of the general SSS ROI displayed minimal high-frequency fluctuations (figure 8*e*,*f*), whereas the final SSS segmentation contained high cardiac frequency activity (figure 8*g*,*h*). These observations confirmed that the data-driven segmentation effectively pinpointed voxels with high cardiac pulsatility in both the arteries and SSS.

## 4. Discussion

This work utilized the pulsatile fMRI signal within large vessels to automatically segment major cerebral arteries and the SSS. Comparisons with TOF segmentations confirmed the accuracy of the data-driven fMRI method in identifying large cerebral arteries. Implementing this technique within the HCP-aging dataset further demonstrated its high reproducibility in between-day scans with the same phase encoding direction. Additionally, spectral analysis confirmed that the algorithm identified voxels with highly pulsatile signals at the cardiac frequency in both arterial and venous regions. The ability to automatically segment large cerebral vessels directly in the fMRI space facilitates future investigations of hemodynamics in these regions. It removes the potential for misalignment when co-registering vascular segmentation imaged outside fMRI space. Lastly, this analytical approach can be applied to any fMRI dataset without prior knowledge, deep learning, or training datasets.

Identifying arteries and veins can enhance two main research streams of fMRI analysis: studies focused on physiological processes and those on neuronal processes. Physiological influences, such as cardiac pulsation, respiration, and vasomotion, affect fMRI signals in the parenchymal tissue [4–7,27–29]. Investigating upstream signals in arteries and downstream signals in venous sinuses can provide insights into parenchymal hemodynamic processes. For example, cerebral transit time has recently been estimated from fMRI signals, where extracting the time series from large vessels is an essential first step in these studies [10,12,30]. In addition to advancing the study of physiological processes, delineating vessel regions and excluding them from grey matter analysis enhances traditional fMRI analysis. Signals from arteries and veins can interfere with nearby neuronal activation (i.e. from neurovascular coupling) measured in grey matter due to partial volume effects. Vascular signal dynamics span a wide frequency range, including low-frequency (0.01–0.1 Hz) [8,31], respiratory (0.2–0.3 Hz) [3,32,33], and cardiac (0.9–1.5 Hz) frequencies [21,32,34]. These signal dynamics are considered noise and can affect the accuracy of functional connectivity measures in grey matter. Zhong *et al.* demonstrated that large arteries and veins significantly impact the surrounding voxels, confounding the functional connectivity assessment [35]. Therefore, automatic segmentation of large vessels in fMRI space and exclusion from grey matter regions is critical for reliable connectivity analyses.

Our data-driven segmentation demonstrates good reproducibility in both arteries and SSS, with the ICC above 0.7. However, slight differences were observed between phase-encoding directions when comparing individual scan volumes, likely caused by image distortions from the echo-planar imaging used in fMRI scans. In scans with AP phase-encoding, the anterior brain is compressed, while the posterior brain is stretched. In scans with PA phase-encoding, the reverse occurs: the anterior brain is stretched, while the posterior brain is compressed. Our artery segmentation focused on the Circle of Willis, primarily localized in the anterior brain. We observed a higher artery segmentation volume in PA phase encoded scans, likely due to stretching the Circle of Willis arteries, which were compressed in the AP phase encoded scans. This is most clearly seen in the MCA in MNI space (electronic supplementary material, figure S9A). The largest portion of the SSS is located near the posterior brain. In our SSS segmentation, we observed a higher artery segmentation volume in the AP phase-encoded scans. This was likely due to the SSS getting stretched in the AP phase-encoded scans and compressed in the PA phase-encoded scans, as illustrated in the sagittal comparison of SSS segmentations in MNI space (electronic supplementary material, figure S9C). These observations further underscore the importance of vessel segmenting in the fMRI space.

Despite strong qualitative agreements between data-driven and ground-truth segmentations, the Dice score for the quantitative comparison was low. Several factors contributed to this, including the co-registration errors between ground-truth segmentation in fMRI space, the complexity and small diameter of cerebral vasculature, as well as partial volume effects. The small size and complexity of the cerebral vasculature mean that even a single voxel misregistration can significantly reduce the dice score (electronic supplementary material, figure S7). Additionally, the dice score is penalized if one segmentation includes more voxels. Due to the fMRI’s high signal dependence on hemodynamics [21,24], voxels partially occupied by blood vessels are more likely to be segmented in the fMRI data-driven segmentation than TOF. As a result, the artery segmentation volume in the data-driven method was around twofold higher than TOF (electronic supplementary material, figure S6). These factors explain the low Dice scores. However, the segmentations still show strong qualitative agreement with ground-truth segmentation localization, and high segmentation probabilities in the MNI spaces corresponding to the Circle of Willis and SSS.

This technique holds certain limitations warranting future exploration. One limitation is that the method requires simultaneous finger plethysmography. While finger plethysmography is an easy signal to acquire, it is only sometimes available in datasets. In future work, techniques can be implemented to generate finger plethysmography signals using fMRI data [23,36] and complete these segmentations without simultaneous finger plethysmography recordings. Another limitation is that the segmentation relies on signals induced by cardiac pulsation, and it is unclear how it will perform in vascular pathologies such as stroke. Additionally, this method was validated in fMRI scans with TRs ≤ 800 ms, faster than commonly used fMRI scan protocols. Although it has not been tested with longer TRs, we expect similar segmentation performance, as Hermes *et al*. have demonstrated that cardiac-aligned fMRI signals are TR-independent [24]. Lastly, this technique identifies vascular regions in fMRI space, and due to fMRI’s inherently large voxel size, the segmentations may contain partial volumes of CSF, grey matter, and white matter.

In conclusion, this work presents a robust, automatic, data-driven tool to segment arteries and veins in fMRI datasets. This approach provides a valuable tool for large vessel segmentations in fMRI, which can be completed independent of vascular angiography scans and, therefore, without registration errors. By offering a reliable method to discern cerebral vasculature, the tool is a crucial resource for improving the accuracy of conventional fMRI connectivity analyses and expanding the feasibility of detailed fMRI-based studies on cerebral physiology.

## Data Availability

The code to complete the data-driven segmentation is available at [37]. The code used in this manuscript is permanently available on Zenodo.org with [38]. The data came from the HCP-Aging 2.0 Release, [39]. The data is available for download through the NIMH Data Archive (https://nda.nih.gov/). Supplementary material is available online [40].
